# Factors Affecting Preserved Renal Volume and Function After Laparoscopic Partial Nephrectomy: A Long-Term 3D Volumetric Analysis

**DOI:** 10.1590/S1677-5538.IBJU.2025.0665

**Published:** 2026-02-20

**Authors:** Onur Kalaycı, Ender Özden, Murat Gülşen, İlkay Çamlıdağ, Ertuğrul Köse, Mehmet Necmettin Mercimek, Yakup Bostancı, Yarkın Kamil Yakupoğlu, Şaban Sarıkaya

**Affiliations:** 1 Samsun City Hospital Department of Urology Samsun Turkey Department of Urology, Samsun City Hospital, Samsun, Turkey; 2 Ondokuz Mayıs University Faculty of Medicine Department of Urology Samsun Turkey Department of Urology, Faculty of Medicine, Ondokuz Mayıs University, Samsun, Turkey; 3 Ondokuz Mayıs University Faculty of Medicine Department of Radiology Samsun Turkey Department of Radiology, Faculty of Medicine, Ondokuz Mayıs University, Samsun, Turkey; 4 Gazi State Hospital Department of Urology Samsun Turkey Department of Urology, Gazi State Hospital, Samsun, Turkey

**Keywords:** Kidney Neoplasms, Laparoscopy, Imaging, Three-Dimensional

## Abstract

**Objective:**

To assess long-term changes in renal volume and function after laparoscopic partial nephrectomy using 3D modeling and to identify key predictors.

**Patients and Methods:**

This retrospective study included 187 patients who underwent laparoscopic partial nephrectomy between October 2012 and January 2023. Patients underwent the same cross-sectional imaging both pre- and postoperatively, with a minimum follow-up of one year. Pre- and postoperative volumes were reconstructed with 3D Slicer software.

**Results:**

The median age of the patients was 58 years. The median Radius-Exophytic-Nearness-Anterior-Location (RENAL) score was 7. The median tumor volume was 15.8 cm³. The median warm ischemia time was 14 minutes, and the median surgical time was 80 minutes.

The mean tumor-free renal parenchymal volume before surgery was 168,87 ± 40,91 cm³, which decreased to a mean operated renal parenchymal volume of 137.6 ± 41.7 cm³ at 5 years postoperatively. The estimated glomerular filtration rate (eGFR) declined from a median value of 90.6 to 75.9 mL/min/1.73 m² over the same period.

The predictors of renal function decline were parenchymal volume loss, age, female gender, diabetes mellitus, and tumor-to-parenchyma contact surface area. Factors affecting parenchymal volume loss included age, RENAL score, comorbidities, Surface-Intermediate-Base (SIB) score, and operative time.

**Conclusions:**

While the most influential factor on renal function in the early postoperative period was the preserved renal volume, diabetes mellitus (DM) emerged as the primary determinant of long-term functional outcomes.

Tumor resection technique and operative time are modifiable factors influencing parenchymal volume preservation. Enucleation-based approaches may enhance parenchymal preservation without compromising oncological outcomes.

## INTRODUCTION

Partial nephrectomy (PN) is the standard treatment modality for patients with cT1 renal tumors when technically feasible, as well as for cT2 tumors in patients with a solitary kidney or chronic kidney disease (CKD) (1).

Contemporary management of small renal masses increasingly emphasizes nephron-sparing strategies with a strong focus on long-term preservation of renal function rather than oncological control alone (2). Regarding functional outcomes, important determinants include warm ischemia time (WIT) and the volume of preserved functional renal parenchyma postoperatively. Previous studies have reported limited data on long-term functional outcomes assessed through kidney volumetry (3, 4).

Three-dimensional (3D) modeling techniques have become integral for obtaining precise and accurate measurements of renal parenchymal volume (RPV) with the help of current technological advancements. Moreover, 3D models facilitate more objective calculations of tumor volume, tumor-parenchyma contact surface area, and bilateral RPV in patients with renal tumors (5).

We hypothesized that preserved renal parenchymal volume is a primary determinant of long-term renal functional preservation after laparoscopic partial nephrectomy, independent of conventional clinical and nephrometry-based factors.

The novel contribution of this study lies in its long-term, three-dimensional volumetric–functional analysis performed in a homogeneous cohort of patients undergoing laparoscopic partial nephrectomy. By minimizing surgical heterogeneity through a single-surgeon, standardized approach, this study provides new insight into the relative impact of preserved renal parenchymal volume and tumor–parenchyma interaction parameters on postoperative renal functional preservation, beyond conventional clinical variables and nephrometry scores.

The primary aim of this study is to evaluate preserved RPV using three-dimensional modeling quantitatively and to examine its association with temporal changes in estimated glomerular filtration rate (eGFR). As a secondary objective, we aimed to identify preoperative and surgical factors influencing preserved RPV and postoperative renal function.

## MATERIALS AND METHODS

This study was conducted with the approval of the Ondokuz Mayıs University Faculty of Medicine Clinical Research and Ethics Committee, dated February 14, 2024 (Decision No: B.30.2.ODM.0.20.08/84-134).

Data from patients who underwent laparoscopic partial nephrectomy (LPN) between October 2012 and January 2023 were prospectively collected and subsequently analyzed retrospectively. A total of 187 patients who had undergone evaluation using the same cross-sectional imaging modality both preoperatively and postoperatively (computed tomography [CT]/magnetic resonance imaging [MRI]) with a minimum follow-up period of one year were included in the study. Patients with a solitary kidney and those with bilateral renal masses were excluded from the study, as it focused on postoperative compensatory functional changes in the contralateral kidney. In the absence of a paired renal unit or in the setting of bilateral surgery, compensatory adaptation cannot be reliably distinguished from the direct effects of surgical intervention, thereby limiting the interpretability of volumetric–functional analyses. Demographic and clinical data such as age, sex, body mass index (BMI), presence of diabetes mellitus (DM), hypertension (HT), coronary artery disease (CAD), CKD (defined as an eGFR below 60 mL/min/1.73 m²), history of smoking, history of prior abdominal surgery, preoperative creatinine levels, and eGFR were recorded.

Preoperative radiological imaging was used to determine tumor size and the RENAL and Preoperative Aspects and Dimensions Used for an Anatomical (PADUA) nephrometry score (6). Additionally, the lateral peripheral fat thickness at the level of the renal vein was measured in alignment with the renal capsule and renal vein. Posterior peripheral fat thickness was assessed by measuring the perpendicular distance from the midpoint of the posterior renal capsule to the posterior abdominal wall at the level of the renal vein. Previous studies have assessed the presence and thickness of perinephric linear soft tissue attenuation (stranding type), and the Mayo Adhesive Probability (MAP) score was calculated based on these data.

A single surgeon performed all surgeries using a previously described technique.

A transperitoneal approach was preferred in all cases, with patients positioned in a modified lateral decubitus position. Pneumoperitoneum was established using a closed technique, and trocar placement was adapted according to patient anatomy. After mobilization of the colon and surrounding structures, Gerota's fascia was incised, and perirenal dissection was carried out to expose the renal hilum. The renal artery and vein were dissected separately and secured with vessel loops.

Tumor localization and resection margins were guided by intraoperative ultrasonography (USG), particularly in endophytic lesions. Tumor excision was performed using cold scissors along the tumor–parenchyma interface, aiming to preserve maximal healthy renal parenchyma and to avoid tumor capsule violation. Selective or global hilar clamping was applied when indicated.

Renorrhaphy was performed using a standardized two-layer technique. The inner layer consisted of absorbable sutures for closure of the collecting system or deep parenchymal defects, while the outer cortical layer was completed using absorbable braided sutures with a sliding-clip technique for parenchymal approximation and hemostasis. After completion of renorrhaphy, vascular clamps were released, and hemostasis was confirmed under reduced pneumoperitoneum pressure. The specimen was retrieved using an endoscopic retrieval bag, and Gerota's fascia was closed with absorbable sutures.

Perioperative variables were evaluated, including operative time, ischemia type and time, intraoperative ultrasonography (USG) use, and the Surface-Intermediate-Base (SIB) score. Complications were classified according to the Clavien-Dindo complication grading system. Postoperative eGFR values were recorded to assess renal function (RF).

Preoperative and postoperative radiological images of the patients were reconstructed into three-dimensional models using the 3D Slicer software (https://www.slicer.org). These models were used to calculate volume and surface area using the software.

Patients with missing clinical, functional, or volumetric imaging data at a given follow-up year were excluded from analyses corresponding to that specific time point. No data imputation was performed. Analyses were conducted on a year-specific basis, including only patients with complete imaging and renal function assessments available for the respective follow-up interval.

### Volume and Surface Area Calculation

**Figure 1:**
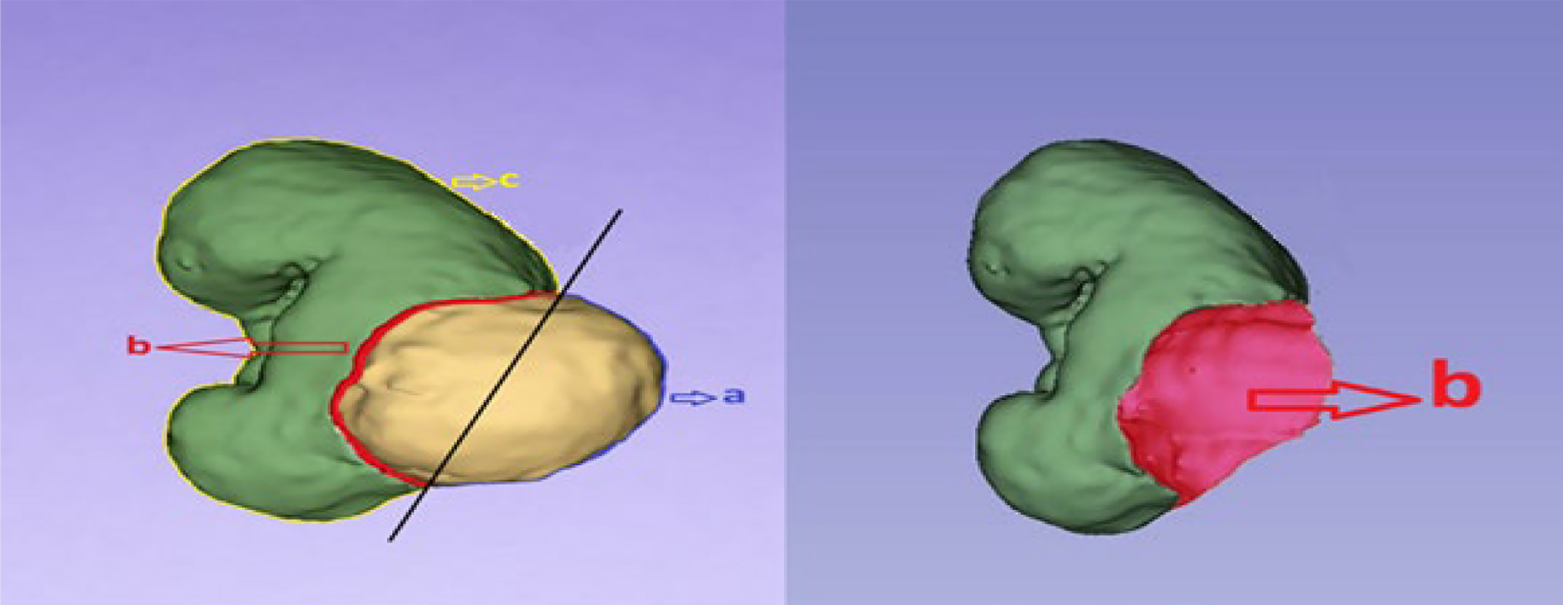
Calculation of the Tumor-to-Kidney Contact Surface Area in Non-Endophytic Tumors

CT/MRI images were processed using the 3D Slicer software to identify the boundaries of the affected kidney, the tumor, and the contralateral kidney to generate 3D reconstructions. The 3D models revealed tumor volume, preoperative healthy RPV (excluding the tumor), contralateral renal parenchymal volume (CRPV), tumor surface area, and tumor-to-kidney contact surface area (as illustrated and described in Figure-1, the tumor-to-kidney contact surface area was calculated).

Suppose we define the exophytic surface area of the tumour as "a", the endophytic surface area as "b", and the outer surface area of the healthy parenchyma as "c". In that case, the 3D Slicer software allows us to calculate the values of "a + c", "a + b", and "b + c". Using these known values, the unknown "b" value, which represents the tumour-to-kidney contact surface area, was calculated using the following formula:

$$\text{b}=\text{tumour}-\text{to}-\text{kidney contact surface area}=\frac{\left(\text{b}+\text{c}\right)-\left(\text{a}+\text{c}\right)+\text{a}+\text{b}}{2}$$

Imaging acquisition protocols were standardized to ensure volumetric accuracy and longitudinal consistency. Contrast-enhanced computed tomography scans were acquired using multiphasic protocols, and tumor assessment and volumetric analyses were performed on the nephrographic phase. Images were obtained with slice thicknesses ranging from 1 to 3 mm and reconstructed using isotropic voxels to enable accurate three-dimensional segmentation. In patients undergoing MRI, contrast-enhanced T1-weighted sequences with comparable slice thickness and reconstruction parameters were used. For each patient, preoperative and postoperative volumetric analyses were consistently performed using the same imaging modality to minimize inter-modality variability.

These measurements were performed as follows: tumor boundaries were delineated in all relevant slices using Hounsfield unit (HU) differences or intensity differences between the healthy parenchyma and the tumor in contrast-enhanced images. Optimal contouring was performed on the most detailed pixel scale in appropriate sequences that demonstrated intensity differences, ensuring precise delineation of tumor borders. Next, the preoperative healthy RPV, excluding the tumor, was measured. The tumor surface area was automatically calculated in square centimeters (cm²) from the 3D models.

### Statistical Analysis

Data were analysed using Statistics Package for Social Sciences version 24 (IBM SPSS®, Armonk, NY) and Number Cruncher Statistical System (ICSS) 11 software. The normality of the distribution was assessed using the Shapiro-Wilk and Kolmogorov-Smirnov tests. The Fisher's Exact Test, Yates Correction, and Pearson Chi-Square Test were used for categorical variables, while multiple comparisons were conducted using the Bonferroni-Corrected Z Test.

For group comparisons, the Independent Samples t-test was employed for normally distributed variables, whereas the Mann-Whitney U test was applied for variables that did not meet the assumption of normality. Linear Regression Analysis was used to examine factors affecting normally distributed dependent variables, while Robust Regression Analysis was applied to those not following a normal distribution.

Results were presented as frequencies (percentages) for categorical variables and as means ± standard deviations or medians (minimum-maximum) for quantitative variables. A p-value < 0.05 was considered statistically significant.

## RESULTS

The demographic and perioperative data of the study are shown in Table-1. Of the patients, 52.9% (n=99) were male, and 47.1% (n=88) were female, with a median age of 58 years (IQR: 48-66). Among the study population, 17.6% (n=33) had DM, 45.5% (n=85) had HT, and 6.4% (n=12) had CKD. Additionally, 35.8% (n=67) had a history of previous abdominal surgery, and 26.2% (n=49) were active smokers.

**Table 1:** Demographic and Perioperative Features of the Cohort.

Demographic Data (n=187)		
**Gender, n (%)**		
	Female	88 (47.1)
	Male	99 (52.9)
Age (years), median (IQR)		58 (48 - 66)
BMI (kg/m²), median (IQR)		28.52 (26.02 - 32.08)
Preoperative eGFR (mL/min/1.73m²), median (IQR)		90.56 (73.99 - 103.16)
Radiological Characteristics (n=187)		
**Tumor Side, n (%)**		
	Left	82 (43.9)
	Right	105 (56.1)
Tumor size (mm), mean ± SD		36.76 ± 13.37
RENAL Score, median (IQR)		7 (6 - 9)
PADUA Score, median (IQR)		8 (7 - 10)
MAP Score, median (IQR)		1 (0 - 3)
Preoperative Volume and Surface Area Measurements (n=187)		
Tumor-Bearing Kidney Volume (cm³), median (IQR)		188.5 (91 - 439)
Tumor Volume (cm³), median (IQR)		15.8 (0.2 - 258)
Preoperative Tumor-Free RPV (cm³), mean ± SD		168,87 ± 40,91
Preoperative CRPV(cm³), mean ± SD		168.87 ± 40.91
Tumor Surface Area (cm²), median (IQR)		35 (3 - 362)
Tumor-to-Parenchyma Contact Surface Area (cm²), median (IQR)		16 (1 - 150)
**Operative Data (n=187)**		
Ischemia Type, n (%)		
	Non-Ischemia	16 (8.6)
	Global Ischemia	161 (86.1)
	Selective Ischemia	10 (5.3)
WIT (min), median (IQR)		14 (11 - 18)
SIB Score, median (IQR)		4 (2 - 5)
Operative Time (min), median (IQR)		80 (65 - 100)
Intraoperative USG Use, n (%)		132 (71)
Estimated Blood Loss (mL), median (IQR)		100 (50 - 160)

Values are presented as median (interquartile range), mean ± standard deviation, or number (percentage), as appropriate.

BMI = body mass index; eGFR = estimated glomerular filtration rate; IQR = interquartile range; SD = standard deviation; RENAL = Radius–Exophytic/Endophytic–Nearness–Anterior/Posterior–Location score; PADUA = Preoperative Aspects and Dimensions Used for an Anatomical score; MAP = Mayo Adhesive Probability score; RPV = renal parenchymal volume; CRPV = contralateral renal parenchymal volume; WIT = warm ischemia time; SIB = surface–ischemia burden; USG = ultrasonography

The tumor was located in the left kidney in 43.9% of patients (n=82) and the right kidney in 56.1% (n=105). The mean tumor size was 36.76 ± 13.37 mm. The median RENAL score was 7 (IQR: 6-9), the median PADUA score was 8 (IQR: 7-10), and the median MAP score was 1 (IQR: 0-3).

Regarding ischemia types, 8.6% (n=16) underwent non-ischemic (off-clamp) surgery, 86.1% (n=161) underwent global ischemia, and 5.3% (n=10) underwent selective ischemia. The median WIT was 14 minutes (IQR: 11-18). The median SIB score was 4 (IQR: 2-5). The median operative time was 80 minutes (IQR: 65-100), with intraoperative USG used in 71% of patients (n=132). The median estimated blood loss was 100 mL (IQR: 50-160).

According to the Clavien-Dindo complication classification, among patients experiencing complications, 4.3% (n=8) had Grade 1 complications, 4.3% (n=8) had Grade 2 complications, and 3.2% (n=6) had Grade 3 complications. Grade 4-5 complications were not observed. Patients were followed for a mean duration of 30 months.

The median preoperative tumor-bearing kidney volume was 188.5 cm³ (IQR: 91-439), the median tumor volume was 15.8 cm³ (IQR: 0.2-258), and the mean tumor-free RPV was 168.87 ± 40.91 cm³. The mean CRPV was 168.87 ± 40.91 cm³. The median tumor surface area was 35 cm² (IQR: 3-362), and the median tumor-to-parenchyma contact surface area was 16 cm² (IQR: 1-150).

The duration of follow-up ranged from 1 to 5 years. The number of patients with complete imaging and renal function data decreased over time, reflecting the retrospective design of the study. Complete follow-up data were available for 187 patients at 1 year, 147 patients at 2 years, 80 patients at 3 years, 36 patients at 4 years, and 12 patients at 5 years. All analyses were performed on a year-specific basis, including only patients with complete data available for the respective follow-up interval.

**Figure 2:**
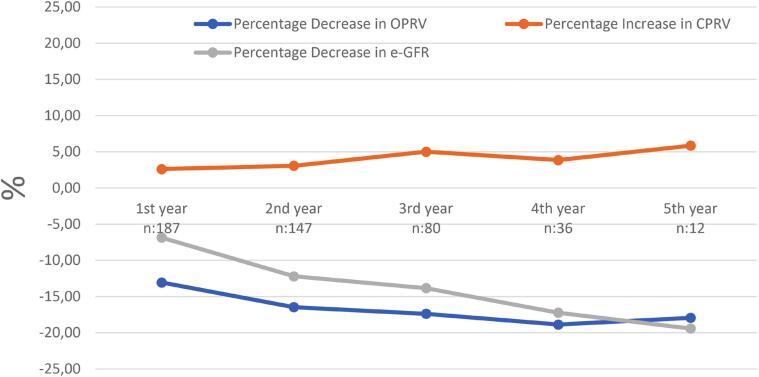
Relationship Between Δ ORPV, Δ CRPV, and Δ eGFR

Preoperative eGFR (mL/min/1.73m²) was 90.56 (73.99–103.16), declining to 84.01 (13.22–121.46) at postoperative year 1, 81.19 (13.79–124.75) at year 2, 81.02 (17.55–118.92) at year 3, 77.66 (15.53–134.94) at year 4, 75.88 (16.81–116.96) at year 5. The operated renal parenchymal volume (ORPV) was measured to be 146 ± 40 cm³ at postoperative year 1, 139.6 ± 40 cm³ at year 2, 139.2 ± 39 cm³ at year 3, 137.1 ± 36.4 cm³ at year 4, and 137.58 ± 41.7 cm³ at year 5. The CRPV was measured to be 173.5 ± 35.9 cm³ at postoperative year 1, 175 ± 35.3 cm³ at year 2, 180.9 ± 36 cm³ at year 3, 178.3 ± 32.9 cm³ at year 4, and 174.17 ± 23.77 cm³ at year 5. The percentage changes in these volumetric data and in eGFR are presented in Figure-2. Robust regression analysis was performed to assess the independent variables influencing ΔeGFR (percentage change in eGFR), which is presented in Table-2. A one-unit increase in age caused a 0.164 decrease in the rate of preserved eGFR in the first postoperative year (p=0.045). Additionally, a one percent decrease in the first-year preserved ORPV led to a 0.197 unit decrease in the first-year rate of preserved eGFR (p<0.001). Furthermore, an increase of one unit in the tumor-to-parenchyma contact surface area resulted in a 0.876 unit decrease in the first-year rate of preserved eGFR (p=0.045).

Longitudinal percentage changes in operated renal parenchymal volume (OPRV), contralateral parenchymal renal parenchymal volume (CPRV), and estimated glomerular filtration rate (eGFR) over a 5-year follow-up period after partial nephrectomy. The graph demonstrates a progressive percentage decrease in OPRV and eGFR, accompanied by a gradual percentage increase in CPRV, indicating compensatory hypertrophy of the contralateral kidney in response to parenchymal volume loss in the operated kidney

n: Number of patients included in the analysis

**Table 2:** Robust Regression Analysis of Factors Associated with eGFR Decline and ORPV Loss Following LPN.

	Δ eGFR			Δ ORPV	
	Year 1 (n:187)	Year 2 (n:147)	Year 3 (n:80)	Year 1 (n:187)	Year 2 (n:147)
	β 0 (95% CI) p	β 0 (95% CI) p	β 0 (95% CI) p	β 0 (95% CI) p	β 0 (95% CI) p
**Patient Factors**					
**Age (years)**	0.16 (0.00 –0.33) **p: 0.045**	0.31 (0.05 – 0.57) p:**0.020**	0.25 (−0.11 – 0.61) p:0.167	0.284 (0.02 – 0.55) p:**0.034**	0.46 (−0.16 – 1.1) p:0.138
**Male Gender**	−2.52 (−5.72 –0.69) p: 0.123	−5.25 (−9.87 – −0.62) p:**0.027**	1.05 (−5.74 – 7.85) p:0.757	−1.88 (−8.73 – 4.96) p:0.59	−7.22 (−22.8 – 8.4) p:0.350
**Body Mass Index**	—	—	—	−0.35 (−0.98 – 0.27) p:0.27	−0.57 (−1.71 – 0.58) p:0.320
**Hypertension**	0.72 (−2.52 – 3.97) p:0.660	2.38 (−2.2 – 6.95) p:0.306	3.21 (−2.85 – 9.28) p:0.293	—	—
**Diabetes Mellitus**	−0.34 (−4.76 – 4.08) p: 0.879	4.22 (−2.02 – 10.46) p:0.183	12.15 (3.24 – 21.07) p:**0.008**	—	—
**Absence of Comorbidities**	—	—	—	−6.17 (−11.1 – −1.25) p:**0.014**	−9.79 (−19.52 – −0.07) p:**0.048**
**Smoking Status**	−1.17 (−4.74 – 2.41) p:0.521	2,66 (−2,64 - 7,96) p:0.322	−1.79 (−9.55 – 5.96) p:0.645	—	—
**Tumor Factors**					
**RENAL Score**	—	—	—	0.33 (−2.12 – 2.78) p:0.790	4.41 (1.72 – 7.1) p:**0.002**
**MAP Score**	—	—	—	−0.82 (−2.48 – 0.84) p:0.331	−1.37 (−5.14 – 2.4) p:0.462
**Preoperative Tumor-Free RPV (cm³)**	−0.04 (−0.08 – 0.01) p: 0.085	0 (−0.07 – 0.06) p:0.993	0.04 (−0.03 – 0.13) p:0.276	—	—
**Tumor Volume (cm³)**	−0.02 (−0.09 – 0.05) p:0.570	−0.04 (−0.16 – 0.1) p:0.451	−0.06 (−0.19 – 0.07) p:0.346	0.01 (−0.10 – 0.12) p:0.898	−0.12 (−0.38 – 0.15) p:0.372
**Contact Surface Area (cm²)**	0.88 (−0.44 – 2.20) p:**0.045**	1.37 (−0.89 – 3.63) p:0.233	1.72 (−1.08 – 4.52) p:0.225	1.64 (−0.65 – 3.94) p:0.159	−0.38 (−3.67 – 2.91) p:0.817
**Surgical Factors**					
**Ischemia Type (Non/Sel)**	—	—	—	−3.85 (−13.26 – 5.55) p:0.418	0.563 (−21.67 – 22.79) p:0.959
**Warm Ischemia Time**	—	—	—	0.01 (−0.59 – 0.62) p:0.960	−0.44 (−1.75 – 0.86) p:0.492
**SIB Score**	—	—	—	2.24 (0.75 – 3.72) p:**0.004**	3.35 (0.53 – 6.17) p:**0.022**
**Operative Time (min)**	—	—	—	0.162 (0.072 – 0.25) p:**0.001**	0.29 (0.10 – 0.48) p:**0.004**
**Intraoperative USG**	—	—	—	1.08 (−3.86 – 6.03) p:0.665	−2.11 (−10.82– 6.59) p:0.622
**Outcomes**					
**Δ ORPV (%)**	0.20 (0.09 – 0.30) p:<**0.001**	0.26 (0.12 – 0.4) p:<**0.001**	0.23 (0.05 – 0.41) p:**0.015**	—	—
**Δ CRPV (%)**	0.03 (−0.14 – 0.20) p:0.739	0.05 (−0.19 – 0.28) p:0.685	−0.17 (−0.49 – 0.15) p:0.293	—	—
**Positive Margin**	—	—	—	−10.61 (−33.84 –12.62) p:0.367	−7.68 (−41.86 – 26.49) p:0.648

A one-unit increase in age led to a 0.310 unit decrease in second-year rate of preserved eGFR (p=0.020). Similarly, a one percent decrease in the second-year preserved ORPV resulted in a 0.259 unit decrease in the second-year rate of preserved eGFR (p<0.001). Additionally, a 5.246 unit greater decrease in the rate of preserved eGFR was observed in female patients compared to male patients in the second year (p=0.027).

A one percent decrease in the third-year preserved ORPV was associated with a 0.229 unit decrease in the third-year rate of preserved eGFR (p=0.015). Furthermore, patients with DM had a rate of preserved eGFR decrease that was 12.154 units higher than those without DM (p=0.008) in the third year.

The robust regression analysis evaluating the independent variables associated with Δ ORPV (percentage change in OPRV) is presented in Table-2. It was determined that for each year increase in age, the first-year rate of preserved ORPV decreased by 0.284 units (p=0.034). Additionally, with an increase of one unit in operative time, the first-year rate of preserved ORPV decreased by 0.162 units (p=0.001). For each unit increase in the SIB score, the first-year rate of preserved ORPV decreased by 2.236 units (p=0.004). Patients without comorbidities had a first-year rate of preserved ORPV 6.166 units higher than those with comorbidities (p=0.014).

As the RENAL score increased, the second-year rate of preserved ORPV decreased by 4.409 units (p=0.002). Additionally, with an increase of one unit in operative time, the second-year rate of preserved ORPV decreased by 0.292 units (p=0.004). Each unit increase in the SIB score resulted in a 3.350 unit decrease in the second-year rate of preserved ORPV (p=0.022). Patients without comorbidities had a second-year rate of preserved ORPV 9.794 units higher than those with comorbidities (p=0.048).

## DISCUSSION

This study identified preserved renal volume as the key modifiable factor influencing eGFR preservation. Tumor resection technique and operative time were additional modifiable factors affecting preserved renal volume. Unmodifiable predictors of eGFR preservation included age, gender, tumor-to-parenchyma contact surface area, and the presence of DM, whereas age, RENAL score, and comorbidities were identified as unmodifiable determinants of renal volume preservation. While the most critical factor on RF in the early postoperative period was the preserved renal volume, DM emerged as the primary determinant of long-term functional outcomes.

Acute kidney injuries secondary to surgery and warm ischemia lead to a renal recovery process. A larger preserved RPV post-surgery correlates with a higher compensatory capacity. Previous studies have emphasised that preserving RPV is the most statistically significant factor in eGFR decline (8, 9). Furthermore, in addition to factors that affect eGFR change by influencing postoperative RPV, other direct determinants of eGFR also exist.

It has been determined that eGFR declines annually as part of the natural ageing process, but the rate of decline slows with advancing age (10). However, postoperative renal functional changes represent a distinct process influenced by surgical and perioperative factors. Similar to our study, studies investigating eGFR decline following PN indicate that the rate of postoperative eGFR decline increases with age (11, 12). Potentially due to more effective compensatory mechanisms in younger patients and increased comorbidities in older individuals.

Furthermore, previous studies have suggested that women indicated a more rapid age-related decline in eGFR than men (13). Studies examining patient and disease characteristics that affect RF after PN have found a greater postoperative eGFR reduction in men than in women (14). However, our study's regression analysis revealed that the postoperative 2-year eGFR decline was 5.24% greater in women than in men. The absence of a gender difference in factors affecting postoperative 1-year eGFR decline, with a difference emerging in the second year, aligns to some extent with studies investigating gender-related eGFR decline in the absence of surgical intervention. This discrepancy highlights the absence of a clear consensus in the literature and suggests that observed sex-related differences may reflect temporal variability rather than a stable, sex-specific pattern of renal functional decline.

DM and HT are well-established risk factors of chronic and end-stage renal disease. Studies examining postoperative eGFR decline in patients with DM and HT have reported debatable outcomes (15, 16). In the literature, studies examining factors affecting RF after PN have demonstrated that baseline RF and DM are significant determinants. Specifically, DM has been highlighted as an independent factor influencing functional outcomes following surgery. These findings highlight the importance of personalised patient management strategies considering the long-term effects of DM on RF (17, 18). In our study, patients with DM exhibited a 12.1% greater eGFR decline than those without DM. At the same time, HT was not statistically significantly associated with RF decline or RPV loss.

Several volumetric analyses in the literature, predominantly conducted in robot-assisted partial nephrectomy cohorts, have emphasized the importance of preserved renal parenchymal volume in determining postoperative renal functional outcomes. These studies have demonstrated that volumetric parameters provide additional insight beyond traditional perioperative metrics. Although the majority of such data originate from robotic series, the volumetric–functional associations observed in our laparoscopic cohort appear to be directionally consistent with these reports. This suggests that the relationship between parenchymal preservation and renal function may be applicable across different minimally invasive approaches, while remaining influenced by technique-specific factors.

Complexity scores such as RENAL and PADUA can predict postoperative RPV changes independently of the surgical technique. Higher scores indicate greater surgical difficulty, making postoperative RF preservation more challenging (6, 19, 20). In our study, higher RENAL scores were associated with lower RPV preservation as well.

Lee et al. compared the predictive capabilities of the RENAL and PADUA scores with tumor contact surface area in assessing RPV changes following PN. They concluded that tumor contact surface area better predicted postoperative RPV changes than these scoring systems (21). Studies examining the relationship between eGFR decline and tumor-to-parenchyma contact surface area have found that patients with a smaller contact surface area experienced less RF decline (22, 23). Our study's regression analysis of independent variables affecting eGFR decline revealed that each 1-unit increase in tumor-to-parenchyma contact surface area resulted in a 0.876% decrease in eGFR (p = 0.045).

Our findings suggest that regression analysis showed that tumor-to-parenchyma contact surface area did not have a statistically significant effect on ORPV changes; however, its significant effect on eGFR decline suggests that even if the RPV is preserved, increased tumor contact surface area may lead to greater nephron loss and perfusion impairment.

MAP score has been linked to functional and oncological outcomes and is useful for predicting difficulties in tumor access and excision (24). However, no studies have examined the relationship between MAP score and postoperative ipsilateral RPV or eGFR change. In our study, no significant association was found between MAP score and eGFR or bilateral RPV changes, possibly due to the high experience of the primary surgeon and the use of laparoscopic USG in complex cases. In support of our findings, a study conducted in our clinic found that using intraoperative laparoscopic USG, specifically in patients with high MAP scores, significantly improved trifecta success rates (25).

Systematic reviews analyzing the effects of WIT on RF following PN have concluded that a limited WIT (<25 minutes) does not negatively impact long-term RF (26). Similar studies investigating different WIT suggest that WIT alone is not the primary determinant of RF loss; other factors play a role (4). Consequently, studies have proposed various cutoff values for WIT, such as 10, 17, or 30 minutes, highlighting the greater impact of non-modifiable factors (e.g., age, DM, HT, BMI, tumor size, preoperative eGFR) on long-term RF (27). Our study's median WIT was 14 minutes, which was not identified as a statistically significant factor affecting RPV change or eGFR decline. This likely reflects that the WIT values in our cohort remained within the literature-defined safe range.

Studies comparing tumor enucleation and standard PN (tumor resection along with a margin of healthy parenchymal tissue) have reported that RF is better preserved in the tumor enucleation group (28, 29). Additionally, in terms of preserved RPV, tumor enucleation resulted in less parenchymal loss compared to standard PN (30). In our study, the SIB score was identified as a significant predictor of RPV change. This may be explained by the greater preservation of parenchymal tissue in enucleation-based PN.

When the SIB score is low, renorrhaphy is more likely to be performed in a relatively avascular area, reducing the amount of healthy parenchyma affected by suturing. This may be one of the factors contributing to better RF preservation. One possible explanation is that a larger tumor–parenchyma contact surface area may predispose to localized or segmental ischemic effects, which could impair renal function even in the absence of substantial measurable parenchymal volume loss. In this context, functional deterioration may occur despite preserved renal volume, reflecting microvascular or regional perfusion changes not captured by volumetric assessment alone.

Beyond surgical technique–specific considerations, preservation of renal function remains a central objective in the management of small renal masses. Minimally invasive alternatives such as thermal ablation have been increasingly adopted in selected patients with small renal masses, largely driven by their favorable renal functional profile and the absence of ischemic insult. Recent reports focusing on risk-adjusted outcomes after radiofrequency ablation have demonstrated acceptable oncological control with limited impact on renal function, underscoring the clinical relevance of parenchymal preservation across different treatment modalities (31).

Operative duration is influenced by patient factors, tumor complexity, surgical method, and the surgeon's expertise. In LPN, these factors influence operative time, leading to prolonged pneumoperitoneum, which can have physiological consequences. Factors contributing to longer operative time, such as tumor complexity, may not only directly impact eGFR and preserved RPV but also have indirect effects by prolonging pneumoperitoneum duration (32). In the present study, operative time emerged as a significant factor associated with postoperative renal outcomes. The reference to pneumoperitoneum should therefore be interpreted as a potential explanatory mechanism underlying prolonged operative duration, rather than as an independently analyzed variable, since the time spent with pneumoperitoneum was not evaluated separately and no direct statistical association was assessed.

Although the operative time in our study was within a reasonable range according to the literature, its effect on ORPV change was considered a consequence of these factors.

The limitations of this study include its retrospective design and the single-centre, single-surgeon experience, which may restrict the generalizability and reproducibility of the findings. However, the single-surgeon experience is also a strength, as it contributes to standardising surgical techniques and postoperative outcomes. Furthermore, volumetric analysis was conducted by a single reader, but minimised interobserver variability and enhanced measurement consistency.

Moreover historically, three-dimensional renal volumetry has been predominantly performed using CT, due to its earlier availability and more widespread clinical integration. Consequently, the majority of volumetric studies in the literature are based on CT datasets. Supplementary analysis demonstrated no statistically significant differences in renal parenchymal or tumor volume measurements between patients imaged with CT versus MRI. This suggests that MRI-based segmentation may serve as a reliable alternative to CT for volumetric evaluation when standardized protocols are applied. While these findings support the use of MRI in this context, larger comparative studies are warranted to further validate its equivalence, particularly for applications involving perfusion or vascular modeling.

Despite these limitations, our study has notable strengths. Advanced three-dimensional volumetric analyses were conducted using a standardised methodology, ensuring the precise assessment of RPV changes. Furthermore, including long-term follow-up data reduces the risk of selection bias, enabling a more comprehensive analysis of RF preservation.

## CONCLUSIONS

Based on the findings of this retrospective, single-center analysis, several clinical and surgical factors appear to influence renal functional outcomes following LPN. Determinants of RF change include patient age, gender, comorbidity burden, tumor-to-parenchyma contact surface area, and loss of ORPV. Renal volume loss was associated with age, comorbidities, RENAL score, operative time, and SIB score.

In selected cases, using the enucleation technique without compromising oncological and functional outcomes is the most modifiable factor for preserving healthy parenchymal volume. Long-term functional outcomes should be carefully monitored in patients with DM, given their potential risk for delayed renal function deterioration.

### Declaration of Generative AI in Scientific Writing

"ChatGPT (OpenAI) was used solely for language editing during the preparation of this manuscript."

## Data Availability

All data generated or analysed during this study are included in this published article
